# Women, Femininity, Indirect and Direct Self-Destructiveness. A Review

**DOI:** 10.1007/s11126-017-9545-4

**Published:** 2017-11-08

**Authors:** Konstantinos Tsirigotis

**Affiliations:** Department of Psychology, Jan Kochanowski University, Piotrków Trybunalski Branch, Słowackiego 114/118 str, 97-300 Piotrków Trybunalski, Poland

**Keywords:** Indirect self-destructiveness, Direct self-destructiveness, Suicide attempts, Biological sex, Sex role, Gender, Femininity, Masculinity

## Abstract

The aim of this work was to review results of research into direct and indirect self-destructiveness in women. Studied projects covered two populations: individuals who attempted suicide and individuals who did not attempt suicide. The Chronic Self-Destructiveness Scale and Bem Sex Role Inventory were used. Intensity of indirect self-destructiveness is lower in women. A probable explanation of the gender paradox in suicides may be the hypothesis that suicides attempted by men more often end in death as men display stronger indirect self-destructiveness. Masculinity and male sex are factors that predispose to indirect self-destructiveness, while femininity and female sex are factors protecting against it. Gender schema opposite to biological sex is significant to intensity of indirect self-destructiveness.

## Introduction

Behaviours causing harm to the subject are generally called self-destructive behaviours. Two basic forms of self-destructive behaviours can be distinguished: direct (open, acute) and indirect (latent, chronic) [1, 2].

### Indirect Self-Destructiveness

Chronic (indirect) self-destructiveness is described as a generalised tendency to undertake behaviours increasing the probability of negative and decreasing the probability of positive consequences for the subject [1]. Indirect/chronic self-destructiveness is also defined as behaviours whose probable negative effect is intermediated by additional factors, while the relationship between the behaviour and harm is perceived as likely. Thus defined indirect/chronic self-destructiveness includes not only the undertaking but also abandoning (commission or omission) of an action; it is a form of self-destruction with an extended distance between an action and consequence [2, 3].

There are several categories of indirectly self-destructive behaviours: transgression and risk, poor health maintenance, personal and social neglects, lack of planfulness, as well as helplessness and passiveness in the face of problems [1, 2, 4].

### Direct Self-Destructiveness

A specific and even tragic form of direct self-destructiveness, because of the gravity and often also irreversibility of its effects, is suicide.

Suicide remains a serious menace to public health around the world. In its individual (personal, intrapsychological) dimension, it is an expression of enormous individual suffering, whereas from the social aspect, it is a tragedy striking the family and friends, as well as depriving the community of its member and benefit he or she might contribute to the society [4].

Suicide attempts are most commonly perceived in terms of direct self-destructiveness. However, suicides and suicide attempts may also be regarded as manifestations or consequences of indirect self-destructiveness, which is a distinctly different form of self-harm than direct self-destructiveness, and an entity different from self-aggression. It is important insofar as both suicide attempts and the intensity of indirect self-destructiveness may still result in a committed suicide. It is not a coincidence that indirect-self destructiveness is referred to as “slow” or “lingering” suicide [4, 5].

### Biological Sex, Gender Role

Biological sex is a set of traits a human is born with, appearing to be obvious and natural. In turn, gender role, and especially the “configuration” of psychological feminine and masculine traits of every human, regardless of his or her biological sex, seems to be less natural and clear.

Money et al. were the first to use the term “sex” with reference to physical traits and the term “gender” referring to the individual’s psychological traits and behaviour [6–8]. Afterwards, Bem formulated her concept rejecting the traditional dichotomous or bipolar masculinity-femininity model by arguing that people have both the traits to a greater or smaller extent, irrespective of their biological sex. The configuration of psychological traits associated with gender (independent of biological sex) results in four gender schema types: sex-typed (feminine women, masculine men), cross-sex-typed or sex-reversed (masculine women, feminine men), androgynous (who to a significant extent possess both feminine and masculine traits), and non-sex-typed or undifferentiated individuals [9–12].

Men are known to exhibit more self-destructive behaviours than women; a majority of research and data, however, concerns direct self-destructiveness. The literature worldwide offers hardly any comprehensive studies into associations between indirect self-destructiveness and gender schema types. Moreover, there are virtually no studies into the gender (sex) differentiation of indirect self-destructiveness intensity considered holistically in individuals who have attempted suicide [4, 13, 14].

The aim of this paper is to review results of research into:

1. Indirect self-destructiveness and its manifestations in women.
2. Differentiation of indirect self-destructiveness with regard to gender roles.
3. Relationships between indirect self-destructiveness and types of gender roles.
4. Suicide attempt methods chosen by women.
5. Direct and indirect self-destructiveness and its manifestations in women after suicide attempts.
6. Relationships between indirect self-destructiveness (and its manifestations) and suicide attempt methods in women.

The studied research projects covered two populations: individuals who had attempted suicide and (healthy) individuals who had not attempted suicide [4, 5, 13–17].

The examination of indirect self-destructiveness used the Polish version of the Chronic Self-Destructiveness Scale (CS-DS) [1] as adapted by Suchańska [2]. Gender roles testing applied the Polish version of the Bem Sex Role Inventory (BSRI) [9, 10] as adapted by Kuczyńska [11, 12].

## Review of Research Results and Discussion

### Indirect Self-Destructiveness, Biological Sex and Gender Roles

#### Indirect Self-Destructiveness and Biological Sex

Women’s scores were lower than men’s for all the categories of indirect self-destructiveness; therefore, it can be assumed that women more rarely and/or less intensely display tendencies and behaviours that, although convenient or pleasant at the time, may prove (physically or psychologically) harmful in the long run [15].

#### Indirect Self-Destructiveness and Psychological Dimensions of Femininity and Masculinity

Significant correlations were found between indirect self-destructiveness categories and masculinity and femininity dimensions. All coefficients between indirect self-destructiveness categories and masculinity were positive, while they were negative between indirect self-destructiveness categories and femininity. Masculinity was associated with indirect self-destructiveness, as well as with transgressive and risky behaviours, and helplessness. It was the converse for femininity: the higher the femininity loading was, the lower the indirect self-destructiveness occurred, and also the lower the personal and social neglects, and lack of planfulness were [15].

Categories of behaviours that constitute ultimate instances of self-destructive behaviours (transgression and risk) in men are connected with masculinity.

The above observations are consistent with those of other studies demonstrating that men are more prone to such risky behaviours as abusing alcohol, not fastening seat belts in cars, performing dangerous jobs, and pursuing criminal activity [15, 18, 19].

Moreover, women run a threefold lower risk of sudden death (accidents, suicides, homicides); they also about three times as rare fall victim to violence and break the law. That divergence is particularly considerable in the case of violent crimes with men more frequently being both perpetrators and victims of such acts [18, 20–22].

The poor health maintenance category is prominent in men. Other studies produced similar results, i.e. men are more prone to avoiding regular contact with physicians [18, 23], while women, irrespective of their condition, find it more difficult to avoid contact with physicians as, for instance, numerous contraceptives are available only if prescribed [18, 24, 25]. Women are more “accustomed” to using health care and more “trained” in that if only due to their necessary regular gynaecological check-ups. Furthermore, women more frequently and willingly seek help in the event of health, life, and/or psychological problems than men, who often consider such a behaviour to be “unmanly” and signify weakness [16, 26]. Thus, those neglects by men have graver health consequences, which is reflected, among others, in the fact that after suicide attempts they require more intensive care than women and those attempts more frequently lead to death [16, 27].

The personal and social neglects category is negatively correlated with femininity, hence femininity seems to be a factor protecting against such problems.

Lack of planfulness also shows negative correlation with femininity, thus femininity is a protective factor in respect of such attitudes and behaviours. Not without significance is the fact that that manifestation of indirect self-destructiveness is lower in women: women most often have to reconcile their careers with running the household and caring for children (which requires organisational skills and planning), and even family planning most often rests mainly on them since most methods and means of contraception are addressed mainly to women [14].

Women also ranked lower than men in helplessness; other studies revealed a relationship between indirect self-destructiveness and a sense of impotence, helplessness and hopelessness [28]. That helplessness is associated with masculinity.

#### Indirect Self-Destructiveness and Types of Gender Schema

Indirect self-destructiveness proved the highest in non-sex-typed and masculine, and the lowest in feminine and androgynous men (predominance of femininity or at least a balance of both the dimensions). Androgynous individuals (women and men) achieved “moderate” scores. In the group of individuals affected by depression, androgynous ones displayed the least intense depressive disorders [29]. What is more, androgynous individuals possessed the greatest psychological resources such as life satisfaction, optimism, sense of self-efficacy and competence [30]. Bem’s (hypo)thesis of a balance of feminine and masculine traits occurring in androgynous individuals as an optimal pattern for mental health may be plausible; in her opinion, the condition for the truly effective human functioning is the full integration of the individual’s masculinity and femininity in a more balanced and complete, genuinely androgynous personality [9, 17, 31]. In the tribes of indigenous North American cultures (Native Americans), there were (and still are) people in whom there is such a harmonious integration of masculine and feminine traits in the psychological and social functioning and behaviour. Native Americans accepted those people: men and women who displayed behaviours appropriate for a different gender and sometimes engaged in sexual relationships with representatives of their own gender. Communities not only accepted those people, but they attributed unique spiritual characteristics to them. They did not consider them to be homosexuals, but beings integrating femininity and masculinity, endowed with the blessing of spirits. Their unusual behaviour, constituting a blend of masculinity and femininity, did not consist in taking “the opposite” gender role, but was rather the equivalent of the “third gender” [18, 32, 33]. European conquistadors called them “berdache”, but the Native American community called for the use of a more appropriate term of “two-spirit people” [34].

#### Women, Men, Indirect Self-Destructiveness and Psychological Dimensions of Femininity and Masculinity

No significant correlation was found between femininity and indirect self-destructiveness in women; in turn, masculinity positively correlated with indirect self-destructiveness, while, in men, femininity negatively correlated with indirect self-destructiveness [17].

The negative correlation between indirect self-destructiveness and femininity in men may suggest that femininity protects men against indirect self-destructiveness.

The positive correlation between indirect self-destructiveness and masculinity in women may indicate that masculinity determines indirect self-destructiveness in women.

Thus, gender role opposite to biological sex – femininity for men and masculinity for women – may seem important to the development of indirect self-destructiveness. The directions of the relationships remain as observed above [15].

The cause of such associations may be explained by the results of other studies. Women characterised by high indirect (chronic) self-destructiveness were found to display tendencies towards negative masculine traits and verbal aggression, but did not possess positive masculine and feminine traits capable of preventing indirect (chronic) self-destructiveness. The results may suggest that coping problems and behavioural difficulties are influenced by similar factors in men and women, but additional negative factors in women, such as “negative masculinity”, may hamper their successful functioning [17, 35].

Femininity protects against, whereas masculinity predisposes to indirect self-destructiveness 13, 37].

### Direct Self-Destructiveness (Suicide Attempts) and Biological Sex

The participants employed several different methods when attempting suicide: abuse of pharmacological drugs (the most prevalent), wrist cutting, hanging, jumping from a height, asphyxia, poisoning and walking into traffic (the least common).

Significant differences concerned abuse of pharmacological drugs and wrist cutting (women), as well as hanging and asphyxia (men). Furthermore, women obtained a higher score with respect to the total number of suicide methods [16].

#### Women, Men and Suicides

In almost the whole world men commit 2–3 times as many suicides as women, but women make more suicide attempts, which is called the “gender paradox” in suicides [16, 27, 36–46]. That gender paradox has also been observed in the study results under consideration: suicide attempts made by women were over three times as common as those made by men. Women also showed higher creativity in inventing methods of attempting suicide. However, despite the considerably larger number of suicide attempts and the number of women making such attempts, women are less often victims of fatal suicides, which indicates that they tend to be the “attempters” and/or “survivors” rather than “performers” of suicides.

#### Women, Men and Suicide Methods

According to some authors, the “lower effectiveness” of women’s suicide attempts may be connected with applied methods. Suicide attempts made by men more often required intensive care and carried a greater risk of death [27], reflecting the more death-oriented intentions of male attempters.

Moreover, most methods reported in the discussed research were characterised by the so-called “softness”, low effectiveness and “feminine” rather than “masculine” attributes. Some authors assume that women “prefer” less effective means, like wrist cuttings or hypnotics abuse, whereas men tend to apply more violent methods such as firearms or jumping from a height [47–49].

#### The Gender Paradox in Suicides Explained (?)

Some theories attempt to explain the gender paradox in suicidal behaviour. The paradox may be accounted for by the fact that men highly rate independence and decisiveness, and consider acknowledging a need for help to be a weakness, thus something to avoid. Women value interdependence, consult their friends, and willingly accept help. They ponder over decisions in the context of relationships, take many things into consideration, and feel freer to change their minds. Treatment seeking, among others, can protect women against fatal suicidal behaviour [16, 50, 51].

Another explanation focuses on methods: men tend to use violent and more lethal methods. Being more prone to aggressive, antisocial and externalising behaviours, they are likely to make more impulsive, lethal, active and determined suicide attempts. A cultural bias should also be taken into account: it could be more difficult for men to admit having problems as that appears to indicate “weakness” [16, 26, 27, 46, 51].

As already mentioned, men display stronger indirect self-destructiveness, both as a generalised behavioural tendency and within its specific manifestations or categories. Hence, yet another plausible explanation of the gender paradox in suicides may be the hypothesis that men’s suicides more commonly result in death as men exhibit stronger indirectly self-destructive tendencies (higher intensity, higher charge of indirect self-destructiveness) than women [15, 17].

### Indirect Self-Destructiveness, Direct Self-Destructiveness (Suicide Attempts) and Biological Sex

The intensity of indirect self-destructiveness and its specific manifestations were higher in individuals who had attempted than in those who had not attempted suicide. Thus, it is not only direct but also indirect self-destructiveness that is more intense in individuals after suicide attempts, its expression, symptom and consequence being the suicide attempt [14, 17].

#### Women, Men, Indirect Self-Destructiveness and Suicide Attempt Methods (Differences)

Although higher in men in the general population [4], indirect self-destructiveness is equally intense in men and women who have attempted suicides. That is important insofar as increased indirect self-destructiveness might be a risk factor and warning sign of suicide attempts [13, 17].

Significant differences in scores were noticed between women and men in four categories of indirectly self-destructive behaviours in individuals who had attempted suicides. Women achieved higher scores on poor health maintenance and lower on three categories: personal and social neglects, lack of planfulness, and helplessness, which was similar in the general population, the difference being that in the latter women scored lower also on poor health maintenance [15]. Women after suicide attempts neglect their health to the greatest extent as compared to the other groups (i.e. men who have attempted suicides and women and men who have not attempted suicides). That is so in spite of the fact that women are, as already mentioned, more “accustomed” to using health care and more “trained” in that [18, 25]. That category of indirect self-destructiveness correlates with recurrent suicide attempts, being a high risk factor for committed suicide [4, 17, 23, 38].

The less intense personal and social neglects in women may stem from the attitude of caring “for everyone and everything” being common in women; hence, care for oneself and others might be assumed to protect women from suicide attempts.

Female gender is a protective factor against lack of planfulness. The phenomenon could be vividly described by saying that men seem to be “careless, cheerful boys” as opposed to women appearing as “constantly worrying work and conscientiousness demons” [17, 52, 53].

#### Women, Men, Indirect Self-Destructiveness and Suicide Attempt Methods (Relationships)

There were many significant correlations but certain differences were found between correlations in the group of women and in the group of men after suicide attempts: some of them occurred only in one group, while others, although present in both, bore a different sign (plus or minus) in each of them.

Relationships between lack of planfulness and abuse of pharmacological drugs and poisoning in women may suggest that a suicide attempt was made using what was within one’s reach. It should be kept in mind that abuse of pharmacological drugs and poisoning are among the least effective suicide methods and are characterised by the lowest self-aggression degree; they are characterised by the lowest degree of body integrity breach, and thus the lowest degree of self-aggression [15, 16, 47, 48]. Therefore, it can be assumed that lack of planfulness in women predisposes to nonfatal suicide attempts that are rather a “manifestation” of problems or a cry for help.

The degree of body integrity breach seems to be quite important in choosing a suicide attempt method. Research indicates that women choose methods that distort their external appearance (e.g. shooting in the head) to a lower degree [54, 55]. That indicates gender determinants resulting not only from psychopathology or suicidology but also from (gender) psychology: even when facing death (or maybe only an attempt at that), women are interested in aesthetic qualities and their own appearance [16].

Attention should be paid to relationships occurring with different signs (plus, minus) in the group of men and in the group of women: personal and social neglects and jumping from a height, asphyxia and recurrence.

Personal and social neglects (more intense in men) correlate positively in men and negatively in women with jumping from a height. It is possible that in that case men display extreme lack of care for all the things important to them; that may be supported by the fact that after a jump, when falling, the individual has little influence on what happens to him or her.^1^ Motivation in women, however, may be different in the case of jumping: abandoning such a decision in women may be influenced by an attitude of caring that is typical of women; after all, it is women who take the most care e.g. of a child from his or her birth (at least at the beginning of the child’s life) and they are also brought up in the spirit of “caring” before they become mothers [13, 17].

## Conclusions

The intensity of indirect self-destructiveness and its manifestations is lower in women. Besides, indirect self-destructiveness and its manifestations correlate mainly positively with masculinity and negatively with femininity.

Femininity is associated with carefulness, cautiousness, attention and planning related to trivial everyday duties as well as matters of the crucial importance to one’s life.

Masculinity is characterised by definitely higher predispositions towards risky and potentially harmful behaviours than femininity.

In particularly difficult situations or those that require specific actions to be taken, predominance of masculine psychological traits allows for giving up, feeling discouraged or lacking willingness to undertake activity that might significantly contribute to ending, solving or eliminating an existing problem or danger.

Masculinity is connected with attitudes and behaviours that prove to be harmful to the subject in the psychological dimension and over time. In turn, femininity predisposes towards good adaptation: the higher the femininity, the lower the indirect self-destructiveness.

Biological sex and gender role together are qualitative variables that differentiate the intensity of indirect self-destructiveness: the highest intensity occurs in non-sex-typed men and the lowest – in feminine ones [17].

Based on the above, it can be assumed that masculinity and male sex are factors that may predispose to indirectly self-destructive behaviours, while femininity and female sex are factors protecting against those [15].

One may hazard a guess that gender differences, even in the case of such a dramatic manifestation of direct self-destructiveness as suicide, may result from higher indirect self-destructiveness in men [15]. Besides women are “attempters” and/or “survivors” of suicide attempts [13, 16].

The intensity of indirect self-destructiveness in women who had attempted suicides (in contrast to the general population) reached the level observed in men who had attempted suicides, which can be a warning sign of suicide attempts in women [17].

The implications of the findings of this work are mainly of preventive and therapeutic character. While analysing the therapeutic aspect, it is necessary to take into account indirectly self-destructive tendencies and behaviours [5]. The evidence for that was offered by a 10-year follow-up of adolescents after suicide attempts: it appeared that 70.50% of the subjects claimed being happy [56]. Furthermore, the optimistic reframing of patients’ negative life events can have therapeutic implications for suicidal activity prevention [57], since pessimism is well-known to be one of the suicide risk factors [58].

Therefore, neither a suicide attempt itself nor its method preclude the possibility of a happy life; that is why it is worth offering individuals after suicide attempts that kind of help and mobilising them to take the opportunity [4, 57, 59, 60]. Both preventive and therapeutic actions can take into consideration the gender-related specificity of the phenomena; it is important to adapt preventive and therapeutic measures to psychological (personality) traits resulting from the individual’s gender. In psychological help or psychotherapy, it may be worth considering the generally favourable impact of femininity, while keeping in mind the not necessarily positive effect of masculinity [14].

As stated above, Bem considers the condition for the truly effective human functioning to be the full integration of his or her masculinity and femininity into a more balanced and complete, genuinely androgynous personality [9, 31], hence femininity may be hypothesised to protect against indirect self-destructiveness [17].

Kelley [35] states that chronic self-destructiveness is not androgynous but rather sex-typed; the results of this study indicate that chronic or indirect self-destructiveness is rather masculine [14, 15, 17].

Based on the above results and findings, one can attempt a description of that phenomenon by stating that femininity reasonably reins in masculinity in its crazy momentum regardless of the biological sex (the intrapersonal perspective) and/or women sensibly rein in men in their crazy momentum (the interpersonal perspective). That consists in the constructive channelling and directing of strong masculinity-related drive and expansiveness [61].

## Footnotes

1 An example of this might be the recent case of a physiotherapist who was going to a patient to carry out rehabilitation (i.e. relieve her suffering) and a (male) suicide who fell on her from the seventh floor, making her disabled (she is now paralysed).
